# Panel testing may not be sufficient to reveal the etiology of suspected genetic epilepsy

**DOI:** 10.1590/1806-9282.20231570

**Published:** 2024-05-27

**Authors:** Josef Finsterer, Fulvio Alexandre Scorza

**Affiliations:** 1 Neurology and Neurophysiology Center – Vienna, Austria.; 2 Unibersidade Federal de São Paulo/Escola Paulista de Medicinal, Neuroscience Discipline – São Paulo (SP), Brazil.

Dear Editor,

We read with interest the article by Baris et al., which is a retrospective, cross-sectional cohort study on 198 pediatric patients with refractory epilepsy and global developmental delay enrolled between July 2018 and July 2021 based on the presence of a pathogenic variant, as assessed by a panel for mutations in genes associated with genetic epilepsy^1^. The most commonly mutated genes in this cohort were the *SCN1A* and *TBC1D24* genes, followed by the *CACNA1A* and *KCNQ2* genes^1^. A pathogenic variant was detected only in three patients (*ALDH7A1, KCNQ2*, and *SCN1A*)^1^. It has been concluded that gene panels support the diagnosis of refractory epilepsy, whereas the undiagnosed conditions remain^1^. The study is impressive, but some points require discussion.

The major limitation of the study is that the pathogenicity of the detected variants was not confirmed by functional or biochemical analysis but only by in silico testing^1^. No population data, computational data, functional data, or segregation data were described. Before a variant is classified as pathogenic, certain criteria as defined by the American College of Medical Genetics and Genomics and the Association for Molecular Pathology system must be met^2^. There are also other classification models that should be satisfied which classify genetic variants as hypomorphic alleles, imprinted alleles, copy number variants, runs of homozygosity, enhancer variants, and variants related to traits^3^.

The second limitation is that a panel testing was performed^1^, which has the disadvantage that new mutated genes not included in the panel may be missed. Panel studies allow only confirm what has been reported previously, while whole exome sequencing (WES) or whole genome sequencing (WGS) allows the detection of a broader spectrum of disease-causing variants.

The third limitation is that panel studies for epilepsy-associated mutations included only genes located on nuclear DNA (nDNA). However, a number of mitochondrial disorders (MIDs) due to variants in mitochondrial DNA (mtDNA) and transmitted through the maternal line manifest phenotypically with epilepsy^4^. Therefore, it is recommended to screen epilepsy patients not only for nDNA but also for mtDNA variants in order not to miss a MID with epilepsy. The best known syndromic MIDs with epilepsy include mitochondrial encephalopathy, lactic acidosis, and stroke-like episode (MELAS) syndrome, Leigh syndrome, Kearns-Sayre syndrome (KSS), and some of the mitochondrial DNA depletion syndromes^5^.

The fourth limitation is that whether the variants occurred in a homozygous or heterozygous distribution was not reported. Knowledge of allele dosage is crucial for predicting outcome and for genetic counselling.

The final limitation is that it was not clarified whether the detected variants were inherited or occurred sporadically. To find out whether the variants were inherited or sporadic, it is imperative to perform family screening for the causative variants. How many of the 198 patients had a positive family history of epilepsy? How many had other first-degree relatives tested for the detected variant of an index patient?

In summary, the interesting study has limitations that put the results and their interpretation into perspective. Addressing these issues would strengthen the conclusions and could improve the status of the study. To clarify the underlying genetic defect in patients with suspected genetic epilepsy, it is imperative to obtain a thorough family history and perform not only panel tests but also WES and, if inconclusive, WGS.

## Data Availability

Data that support the findings of the study are available from the corresponding author.
